# Pentoxifylline and Tocopherol for the Management of Medication-Related Osteonecrosis of the Jaw (MRONJ): A Retrospective Clinical Audit

**DOI:** 10.3390/antibiotics15030280

**Published:** 2026-03-10

**Authors:** Niccolò Lombardi, Virina Basta, Chiara Morelli, Giulia Ghidini, Giovanni Lodi, Elena M. Varoni

**Affiliations:** Department of Biomedical, Surgical and Dental Sciences, Università degli Studi di Milano, Via Beldiletto 1, 20142 Milan, Italy; niccolo.lombardi@unimi.it (N.L.); virinabasta123@gmail.com (V.B.); chiara.morelli1@unimi.it (C.M.); giulia.ghidini@unimi.it (G.G.)

**Keywords:** antibiotics, pentoxifylline, tocopherol, osteonecrosis, sequestrectomy, bisphosphonates

## Abstract

**Background/Objectives**: Medication-related osteonecrosis of the jaw (MRONJ) is a challenging complication in patients receiving antiresorptive therapy. Management strategies range from conservative pharmacological approaches to extensive surgical resection of necrotic bone. This clinical audit retrospectively evaluated the clinical outcomes of patients undergoing sequestrectomy for MRONJ, comparing those treated with antibiotics alone with those receiving antibiotics in combination with the pentoxifylline–tocopherol (PENTO) protocol. The PENTO protocol was introduced at our institution in 2021 and has since been routinely prescribed for all MRONJ patients. **Methods**: We analyzed 92 MRONJ sites treated with sequestrectomy. Conservative management consisted of antibiotic therapy, with or without adjunctive PENTO (pentoxifylline 800 mg/day and tocopherol 800 IU/day, administered both preoperatively and postoperatively). The primary outcome was healing at three months post-surgery, while the secondary outcome was disease recurrence during longer-term follow-up. **Results**: Complete healing was achieved in 56 of the 92 sites, with a mean follow-up of 9.98 ± 12.76 months among healed cases. No statistically significant differences in healing rates were observed between the PENTO and antibiotic-only groups. The overall recurrence rate was 12.5%, with no significant difference between the groups. **Conclusions**: Overall, surgical management of MRONJ resulted in favorable outcomes in a substantial proportion of patients. Within the limitations of this retrospective clinical audit, the addition of PENTO to antibiotic therapy appeared generally well tolerated, but could not result in a significant improvement in healing rates or reduction in recurrences, compared with antibiotic therapy alone, in this patient cohort.

## 1. Introduction

Medication-related osteonecrosis of the jaw (MRONJ) is a rare and debilitating multifactorial condition often associated with antiresorptive and antiangiogenic agents, used for osteometabolic conditions such as osteoporosis, Paget’s disease, and algodystrophy, malignancy-related hypercalcemia, or in the treatment of bone lesions associated with multiple myeloma or with metastatic malignancy from solid tumors [1,2,3,4].

According to the American Association of Oral and Maxillofacial Surgeons (AAOMS), the diagnosis of MRONJ requires the simultaneous presence of three criteria: current or previous treatment with antiresorptive or antiangiogenic agents; visible or probeable exposed bone in the maxillofacial region persisting for more than eight weeks; and absence of a history of radiotherapy or metastatic disease involving the jaws [1,2,5,6,7].

The diagnosis of MRONJ is primarily clinical; however, complementary diagnostic examinations are valuable for confirming the diagnosis and assessing disease progression, including orthopantomography, computed tomography, and magnetic resonance imaging [8].

The AAOMS recognizes three main classes of medications associated with MRONJ: bisphosphonates, RANK-L inhibitors, and antiangiogenic medications [1,2]. Bisphosphonates, derived from inorganic pyrophosphates, inhibit bone mineralization and resorption and promote the apoptosis of osteoclasts [9]. Denosumab, a monoclonal antibody, binds to RANK-L, inhibiting the formation and function of osteoclasts, while antiangiogenic drugs inhibit the formation of new blood vessels, possibly leading to ischemia and hypoperfusion [4,7]. To evaluate the risk of MRONJ development, it is essential to consider both the route of administration and the duration of drug treatment, as the risk is dose- and time-dependent, increasing with higher cumulative doses and longer treatment duration [2,10].

Among patients diagnosed with MRONJ, several studies report that tooth extraction was a triggering event in 62% to 82% of cases [10]. The presence of pre-existing periodontal or periapical infection in patients treated with antiresorptive or antiangiogenic medications is also a well-recognized risk factor for the development of MRONJ. MRONJ occurs more frequently in the mandible (75%) than in the maxilla (25%); however, in some cases, both jaws may be involved (4.5%) [2,10]. The AAOMS staging system for MRONJ, first introduced in 2009 and updated in 2022, classifies the disease into four stages based on clinical and radiographic findings [6,8,10]. Stage 0 includes patients without exposed necrotic bone but with nonspecific clinical or radiographic signs. Stage 1 is characterized by exposed necrotic bone or a fistula probing to bone in asymptomatic patients without infection. Stage 2 involves exposed necrotic bone or a fistula probing to bone with infection and clinical symptoms. Stage 3 represents advanced disease with exposed necrotic bone and infection associated with major complications, such as extension beyond the alveolar bone, pathologic fracture, extraoral fistula, oroantral/oronasal communication, or extensive osteolysis.

The treatment of MRONJ is often challenging and there is no standardized therapeutic approach [1,11]. According to the AAOMS, the main goals of treatment are to eliminate pain, control infection, and minimize the occurrence or progression of MRONJ [11]. The choice between surgical and non-surgical therapy must be specific and adapted to the needs of each patient. A multidisciplinary approach is essential, and collaboration between oncologists, rheumatologists, dentists, and oral and maxillofacial surgeons is essential for a comprehensive evaluation of the patient [11]. Surgical treatments for MRONJ range from conservative procedures, such as bone debridement and sequestrectomy, to more invasive approaches, including marginal or segmental mandibulectomy, with or without jawbone reconstruction, when indicated; outcomes are particularly favorable when surgery is performed in the early stages [12,13,14,15]. Non-surgical strategies may be beneficial at any stage, particularly when comorbidities contraindicate surgical intervention or when comorbidities associated with surgery could severely impact on patient’s quality of life. Non-invasive procedures include medical treatment, negative pressure wound therapy (NPWT) [16], the use of pentoxifylline (associated or not with tocopherol) [17,18], Er: YAG laser ablation, and Nd: YAG/diode laser biostimulation [19,20] and administration of teriparatide [21,22].

The combined use of pentoxifylline and tocopherol (PENTO) is an anti-fibrosis and anti-oxidation protocol for the treatment of osteoradionecrosis (ORN) based on a “radiation-induced fibrosis” theory proposed by Delanian [23]. Pentoxifylline is a methylxanthine derivative, used for the treatment of vascular disorders, which is able to increase microvascular perfusion and to act against some inflammatory mediators, including TNF-α [24]. Tocopherol is a methylated phenolic compound that is part of the vitamin E group, and it acts as an antioxidant agent. In combination with pentoxifylline, a positive synergistic effect has been observed on the progression of fibrotic and inflammatory lesions resulting from radiotherapy [24]. Hence, the recent literature suggests a promising role of PENTO as pharmacological therapy in the prevention and management of ORN as well as for the management of MRONJ, to gradually promote bone sequestration, thus facilitating the following surgical phase [25,26,27]. In case of MRONJ, in particular, pentoxifylline and tocopherol can improve microcirculation, reduce oxidative inflammation, and promote tissue regeneration [25,27]. PENTO therapy has demonstrated both subjective and objective benefits, notably in pain reduction and the promotion of new bone formation [17,18,25,28].

This study aimed to retrospectively assess, through a clinical audit, the success and recurrence rates in a cohort of MRONJ patients, comparing those treated with the PENTO protocol plus antibiotics to those receiving antibiotics alone. In our Institution, the adjunctive use of PENTO was introduced in 2021 to promote bone sequestrum formation prior to the subsequent conservative surgical phase, with the ultimate goal of minimizing the need for extensive resections. The treatment protocol, based on recent literature, primarily involved antibiotic therapy combined with local surgical intervention, with follow-up continuing until bone sequestrum formation [10,29,30,31,32].

## 2. Results

The initial cohort included 125 patients diagnosed with MRONJ. Of these, 86 patients (70 females, 16 males; mean age = 72.22 ± 13.17 years, range 30–95 years) met the inclusion criteria and were enrolled in the study, while 39 were excluded for the following reasons: history of head and neck radiotherapy (number of patients, n = 30) and absence of exposure to medications associated with MRONJ risk (n = 9). These 86 patients underwent a total of 92 sequestrectomy procedures, with 6 patients receiving two separate interventions in different areas of the maxillofacial region.

A total of 85 patients had at least one month of postoperative follow-up (one patient showed a follow-up < 1 month). The mean follow-up from the first diagnostic visit to the last visit was 9.16 ± 11.19 months (range: 0–60 months). At three months, follow-up data were available for 65 patients, accounting for 69 MRONJ sites.

**Figure 1:**
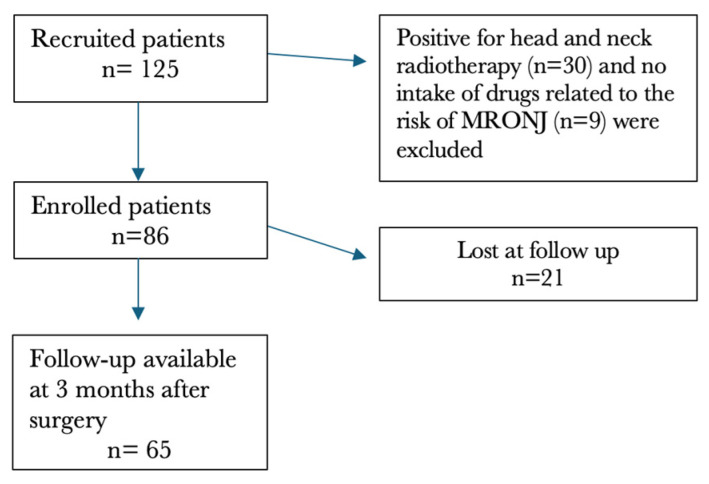
Flow chart of enrolled study patients.

Figure 1 provides a flow chart of included patients with 3-month follow-ups available.

The primary conditions requiring antiresorptive therapy were predominantly oncological (63.95% of patients), followed by osteometabolic disorders (34.88% of patients). Among the 86 patients, the drugs most frequently associated with MRONJ were denosumab, administered to 34 patients (39.53%), and zoledronate, used in 25 patients (24.42%). Patients also received various concomitant therapies, including chemotherapy (e.g., methotrexate, eribulin, paclitaxel), corticosteroids (e.g., dexamethasone, prednisone, deltacortene), hormone therapy (e.g., leuprorelin), monoclonal antibody therapy (e.g., trastuzumab, brigatinib, lenalidomide, ribociclib), or combinations of these drugs (e.g., tamoxifen plus trastuzumab). However, 48.85% of patients did not receive any additonal concomitant drug therapy which may further represent a risk factor, together with antiresorptive therapy, for the onset of MRONJ.

Clinically, MUCONN scores below 5 indicate a low risk of MRONJ and accounted for the majority of patients in both treatment groups, followed by those with moderate-risk MUCONN scores. High-risk MUCONN scores were rare overall, limiting their impact on comparative interpretation between the two treatment strategies. In particular, more than half of the overall cohort (54.65% of patients) fell within the low-risk range (scores 1–5), 40.70% of patients fell within the moderate-risk range (scores 6-10), while high-risk scores (11–15) were uncommon, occurring in only three patients overall (3.49%) (Table 1). In the low-risk category, 34 patients treated with the PENTO protocol and 13 patients in the antibiotic group had MUCONN scores between 1 and 5. Moderate-risk scores (6–10) were observed in 25 patients receiving PENTO and in 10 patients treated with antibiotics. Notably, all patients classified in the high-risk category (MUCONN 11–15) were treated with the PENTO protocol, with no patients in the antibiotic group represented in this range. 

The most prevalent MRONJ stage was stage 2, observed in 41 patients (47.68%). Stage 1 was recorded in 32 patients (37.21%), stage 3 in 11 patients (12.79%), and stage 0 in 1 patient (1.16%); in one case (1.09%), the stage was not specified. The mandible was the most frequently affected site, involved in 56 patients (65.12%). The events suspected to be associated with MRONJ onset included dental extraction in 29.07% of cases, implant placement in 29.07%, and spontaneous occurrence in 41.86% of patients. Spontaneous exfoliation of bone sequestra occurred in 5.81% of the cohort, of whom four out of five patients had received tocopherol and pentoxifylline therapy. The evolution time of bone sequestration (i.e., time from first visit up to bone sequestrum formation), evaluated in 21 patients for whom the data were available, was 148.61 days (SD ± 130.41; range: 19 to 480 days).

Patient demographic and clinical data are summarized in Table 1.

Of the 86 patients included in the cohort, 62 (72.1%) received the combined PENTO protocol with antibiotic therapy (e.g., amoxicillin, amoxicillin/clavulanic acid, metronidazole, cefixime); of them, 56 were treated after 2021, while 6 in 2019–2020. The remaining 24 patients (27.9%) were treated with antibiotics alone; of them, 16 were treated in 2019–2020, while 12 were treated after 2021. The most prescribed antibiotic was amoxicillin, administered as one tablet every 8 h (three times daily) from three days before surgery until two weeks postoperatively, or adjusted according to clinical conditions. Considering patients with 3 months follow-up (n = 65), in particular, 43 (66.15%) patients received PENTO and antibiotics, accounting for 47 MRONJ sites, while the remaining 22 (33.85%) received only antibiotics, accounting for 22 MRONJ sites. Baseline demographic and clinical characteristics of patients treated with PENTO (plus antibiotics) or with antibiotics alone are summarized in Table 2, including MRONJ stage, MUCONN score distribution, antiresorptive therapy, and age. Considering our data, the PENTO group had slightly higher percentages of patients with advanced disease compared to the antibiotics-only group, i.e., Stage III was 14.5% in the PENTO group and 8.3% in the antibiotic group, although with no statistically significant difference between the two groups (Fisher’s exact test, *p* = 0.48).

### 2.1. Primary Outcome

#### Clinical Healing of MRONJ

A patient was defined as healed if the clinical conditions, i.e., absence of bone exposure, oral mucosal inflammation, infection, or clinical drainage, were maintained for at least 3 months following sequestrectomy or spontaneous exfoliation of necrotic bone. At 3 months, follow-up data were available for 65 patients (62 patients in the PENTO group; 24 patients in the antibiotics group), accounting for 69 MRONJ sites.

Considering MRONJ sites as statistical unit for the primary outcome, the clinical healing at 3 months was observed, overall, in 56 out of 69 sequestrectomies for which the follow-up of at least 3 months was available (81.15%).

Among the MRONJ sites in patients who received PENTO and antibiotics, 37 out of 47 achieved complete healing (78.72%), while in the MRONJ sites of antibiotic-only group, 19 out of 22 achieved complete healing (86.36%). In the PENTO-treated group, patient healing rates were comparable across stages (76.9% in stage I patients, 88.9% in stage II, and 77.8% in stage III), with no statistically significant differences among stages (Fisher’s exact test, *p* > 0.05). When comparing patient subgroups by antiresorptive drug type (denosumab vs. bisphosphonates) within the PENTO group, healing occurred in 78.6% of those receiving denosumab (22/28) and 85.2% of those receiving bisphosphonates (29/34), with no significant difference between the two groups (Fisher’s exact test, *p* > 0.05).

**Figure 2:**
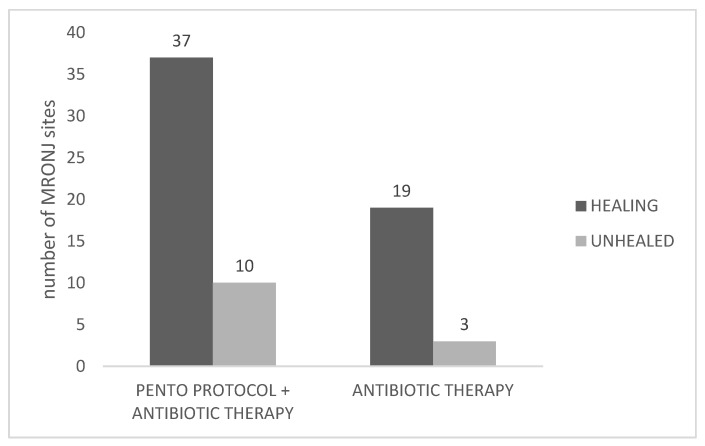
Primary outcome: clinical healing of MRONJ sites.

Figure 2 shows the primary outcomes.

No statistically significant difference in clinical healing was observed between the group receiving the PENTO protocol combined with antibiotic therapy and the group receiving antibiotic therapy alone (χ^2^ = 0.57, *p* = 0.45). The odds ratio of 0.58 suggested lower odds of healing in the PENTO group.

Overall, among the non-healed MRONJ sites (n = 13), only two experienced clinical worsening, belonging to the PENTO+ antibiotics group (Table 3). These two patients showed disease progression two months after surgery and were classified as stage 2 and stage 3, respectively; both of them had mandibular MRONJ.

### 2.2. Secondary Outcome

#### 2.2.1. Recurrences

Recurrences were observed in 7 MRONJ sites, out 56 healed MRONJ sites (12.5%), in five patients. The mean time to recurrence onset was 8.0 ± 11.49 months, with a range from 3 to 34 months. Patients who experienced recurrence mainly received denosumab.

**Figure 3:**
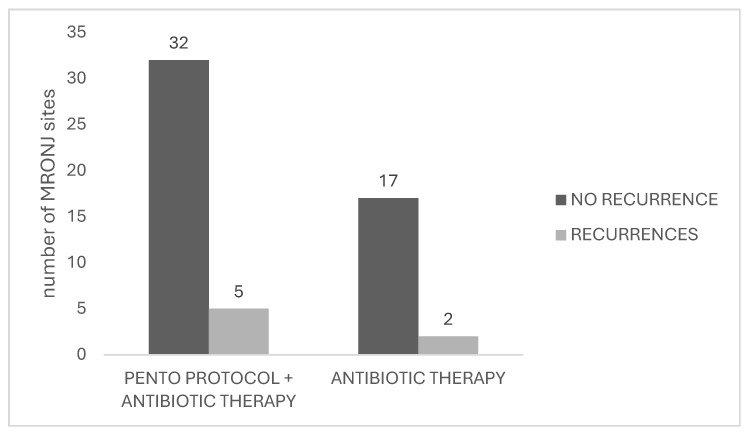
Secondary outcome: recurrences of MRONJ.

In total, 5 out of 37 healed MRONJ sites in the PENTO group experienced recurrence (13.5%), while two recurrences out of 19 healed MRONJ sites were observed in the antibiotic group (10.5%) (Figure 3), without statistically significant difference (χ^2^ = 0.095, df = 1, *p* = 0.76). The odds ratio of 1.33 suggested a slightly higher odds of recurrence in the PENTO group compared with the antibiotic-only group.

#### 2.2.2. Adverse Events

This retrospective review of the patients’ clinical records revealed that none documented any adverse effects. However, this finding should be interpreted with caution, as the retrospective design relies on the completeness and accuracy of clinical documentation and may underestimate adverse effects that were not systematically recorded.

## 3. Discussion

MRONJ is a pathological condition that most frequently affects females, elderly individuals, and patients receiving antiresorptive therapy for oncologic indications [33,34,35]. This complication represents a significant clinical challenge for dental practitioners, especially in terms of management. The treatment of MRONJ ranges from conservative pharmacological management with antibiotics and antiseptics to extensive surgical resection of necrotic bone. According to previous studies [13,34,35,36,37], an early surgical approach with adequate resection margins and primary wound closure may lead to improved clinical outcomes, including complete mucosal healing without signs of infection at six months and radiographic evidence of healing one year after treatment. Since pharmacological therapy alone rarely leads to lesion healing, even in the early stages [15], Khan and colleagues [38] have recommended surgical resection with tension-free primary closure.

In our clinical audit, we evaluated the effect of the PENTO protocol combined with antibiotic therapy compared with antibiotic therapy alone in patients undergoing sequestrectomy for MRONJ. Independently from the PENTO protocol, overall, the complete healing was achieved in 56 of the 92 MRONJ sites (60.87%) treated with sequestrectomy, also showing a mean follow-up of 10 months. These results support the effectiveness of surgical management in achieving favorable outcomes in a substantial proportion of patients. When comparing treatment protocols, no statistically significant differences were observed between patients receiving the PENTO protocol in combination with antibiotic therapy and those treated with antibiotics alone in terms of healing rates.

Considering recurrence rates, overall, 7 MRONJ sites out 56 previously healed MRONJ sites (12.5%) experienced recurrences. This rate is consistent with previous studies, which described recurrence rates following surgical management of MRONJ ranging from 11.8% to 30.4% [32,39,40]. No statistically significant difference was observed between the PENTO and antibiotic-only groups. 

No side effects of PENTO protocol were mentioned in the clinical records; however, previous studies found that pentoxifylline may cause a range of adverse effects, most commonly involving the gastrointestinal and nervous systems. Frequently reported side effects include nausea, vomiting, abdominal discomfort, bloating, and diarrhea, as well as dizziness and headache. Vasodilatory effects such as flushing are also common. Less frequently, cardiovascular and respiratory adverse events have been reported, including chest pain, arrhythmias, hypertension or hypotension, tachycardia, and dyspnea [41,42]. To note, safety evaluation was based only on retrospective chart review, with potential underreporting bias.

The main limitations of this study include the retrospective design, based on the review of medical records, which may have resulted in incomplete or non-uniform data collection and limited control over potential confounding variables, including the duration of PENTO administration and antibiotic therapy that could vary according to individual patient characteristics and clinical conditions, despite the availability of a standardized treatment protocol. Moreover, patients receiving the PENTO protocol may have represented those with more severe disease; adjustment for MUCONN scores could be considered in future analytic studies designed to assess independent associations. This was also a single-center study conducted in a university-based referral setting, which may have introduced selection bias and limited the external validity and generalizability of the findings to other clinical contexts or patient populations. Furthermore, although representative of a specialized clinical unit, the sample size may have been relatively limited; therefore, the absence of statistically significant differences between groups may reflect limited statistical power rather than true equivalence. Along these lines, the study population was also clinically heterogeneous with respect to underlying conditions, type and duration of antiresorptive therapy, MRONJ stage, and concomitant oncological treatments, potentially affecting the robustness of stratified analyses. Variability in follow-up duration among patients may have led to an underestimation of late recurrences or long-term complications. A potential temporal bias should also be acknowledged, as PENTO was routinely implemented only after 2021, and improvements in outcomes may therefore partially reflect contemporaneous changes in clinical practice, experience, or follow-up duration rather than the treatment effect alone.

Overall, our findings that the PENTO protocol may represent a well-tolerated therapeutic adjuvant strategy in MRONJ patients receiving bone sequestration intervention, however it needs to be further explored to evaluate the efficacy. Further prospective, randomized, and long-term studies on larger cohorts are warranted to confirm these results and to better elucidate the underlying mechanisms responsible for the clinical efficacy of the PENTO protocol.

## 4. Materials and Methods

### 4.1. Patients

#### 4.1.1. Study Design and Patient Population

A clinical audit was conducted by reviewing the medical records of patients diagnosed with MRONJ who were referred to the Oral Medicine Unit at ASST Santi Paolo e Carlo, University of Milan, between July 2019 and June 2025. MRONJ cases were staged at the time of diagnosis according to the updated classification criteria established by the American Association of Oral and Maxillofacial Surgeons (AAOMS) in 2022 [10]. This report was written in accordance with the Standards for Quality Improvement Reporting Excellence (SQuIRE) guidelines.

#### 4.1.2. Eligibility Criteria

Inclusion criteria consisted of patients diagnosed with MRONJ at stages 0 to III, according to the AAOMS classification system [10,43]. All included patients had undergone an initial phase of pharmacological management, followed by surgical intervention aimed at removing necrotic bone sequestra [10,29,30,31].

Exclusion criteria were as follows: a history of radiation therapy to the jaws or the presence of metastatic disease involving the jaws; absence of a pharmacological history consistent with the development of MRONJ.

### 4.2. Data Collection

Clinical and demographic data collected for each patient included: age, gender, age at MRONJ onset, MUCONN score [44,45], primary condition requiring treatment with antiresorptive medications, type of bisphosphonate used, combined oncological treatments, MRONJ stage [10], formation of bone sequestra, timing and localization of exposed bone, date(s) of surgical intervention, pharmacological treatments administered, number of recurrences, length of follow-up, and etiology of the osteonecrosis. The duration of therapy was defined as the time from the initiation of antiresorptive treatment to the patient’s first visit to our clinical unit.

To evaluate the potential correlation between patient systemic conditions and treatment outcomes, we applied the Modified UCONN Score (MUCONN), as described by Reich et al. [44]. This prognostic scoring system accounts for various comorbidities; each assigned a weighted value based on its severity and potential impact on the development and progression of MRONJ [45]. Specifically, osteoporosis scores 1 point, diabetes mellitus 2 points, oral steroid administration 2 points, breast and prostate cancer (among other malignancies) 3 points, and chemotherapy 5 points. For each patient, the total MUCONN score was calculated by summing the weighted values of applicable comorbidities, providing an index to assess individual risk and prognosis [45].

#### MRONJ Intervention

Based on updated reviews of both non-surgical and surgical therapies, the management of MRONJ was based on pharmacological approach before the conservative surgical intervention intended to remove bone sequestrum [10,29,30,31]. The sequestrum had to be clinically or radiographically evident.

Following the internal protocol of the hospital unit, all patients were prescribed antibiotic therapy with amoxicillin (or amoxicillin combined with clavulanic acid) at 3 g/day, administered as 1 g every 8 h, starting 3 days before surgery and continuing for at least 10 days postoperatively [46]. In case of penicillin allergy, clarithromycin 1 g/day was used (500 mg every 12 h starting 3 days before surgery and continuing for at least 10 days postoperatively). If monotherapy was insufficient, metronidazole 500 mg twice a day was added to create a combined antibiotic regimen. Additionally, antiseptic therapy was prescribed, consisting of 0.12% chlorhexidine mouthwash and 1% chlorhexidine gel applied directly to the exposed necrotic bone, starting 3 days before surgery and continuing for at least 3 weeks postoperatively [31]. Each patient underwent dental scaling and local antiseptic treatment for four weeks to one week before surgery. 

*Surgical intervention*—The surgical procedure (sequestrectomy) involved removal of the necrotic bone under local anesthesia and elevation of a full-thickness mucoperiosteal flap, extended to fully expose the necrotic area and reach beyond it to disease-free margins. Teeth involved in the necrotic process were extracted; curettage and bone remodeling were performed until viable bleeding bone was observed, indicating healthy tissue. In cases where the bone sequestrum showed evident mobility, the underlying granulation tissue was left in place to cover and protect the remaining healthy bone component, thereby reducing the risk of new MRONJ recurrence. Flaps were repositioned and sutured with absorbable sutures after periosteal-releasing incisions to achieve primary closure and maximize vascular supply to the underlying bone. This approach helps reduce the risk of postoperative infection at the surgical site. In the postoperative period, patients continued both antibiotic and antiseptic therapies to support healing and prevent infection.

*PENTO protocol*—In our institution, the PENTO protocol was introduced in 2021. After its introduction, PENTO was not systematically prescribed to all eligible patients, although it was adopted in most cases; its use was guided by clinical judgment and additional clinical criteria, the latter including the presence of active anticancer treatment, which required confirmation of protocol safety evaluation by the treating oncologist. From 2021 onward, PENTO protocol was prescribed to MRONJ patients according to a standardized regimen consisting of pentoxifylline 800 mg/day and tocopherol 800 IU/day, administered 4 weeks before surgery and 8 weeks after surgery; however, the duration of both pre- and postoperative therapy were tailored to the clinical scenario [18,25,27,42].

### 4.3. Outcomes

Outcome assessments were performed by multiple clinicians, under standardized internal criteria.

#### 4.3.1. Primary Outcome: Clinical Healing of MRONJ

The criteria used for assessing the clinical healing are described in Table 4 (adapted from [33]):

#### 4.3.2. Secondary Outcome: Rate of MRONJ Recurrence

Consistently with previous literature [32,39,47] recurrence was defined as the occurrence of exposed necrotic bone, or bone that could be probed through a fistula in an area that had previously demonstrated long-term healing, with or without radiographic evidence of architectural bone changes, persisting for more than 8 weeks.

### 4.4. Statistical Analysis

The statistical unit for outcome assessments was the number of MRONJ sites, since individual patients could present with multiple anatomically and clinically distinct lesions that were spatially separated, managed independently, and followed different therapeutic courses. This approach was consistent with prior lesion-based MRONJ studies [15,35].

Means and standard deviations were calculated for continuous variables; Kolmogorov–Smirnov test of normality was applied, and data were normally distributed. A *t*-test was used to compare means between two unpaired samples.

For categorical variables, extracted data were expressed as percentages, and statistical analyses to identify significant differences were performed by applying the χ^2^ test using the online Graphpad statistical software (GraphPad Prism 10.6 Software^®^, San Diego, CA, USA).

Odds ratios were also calculated using the online MedCalc Software Ltd. statistical software (MedCalc 23.4.2 Software Ltd., Ostend, Belgium). Effect estimates, including odds ratios, were reported together with their corresponding 95% confidence intervals (CI) to quantify the magnitude and precision of the observed associations. As this study was conducted as a clinical audit focused on describing local practice, MUCONN was not incorporated into adjusted analyses, and no multivariable modeling was undertaken. Statistical significance was set at *p* ≤ 0.05.

### 4.5. Ethical Approval

This is a clinical audit, which follows institutional and national regulations for the improvement of quality of care. In accordance with the principles of the Declaration of Helsinki (2013 revision), the audit was performed using routinely collected clinical data, with no deviation from standard care and with appropriate measures taken to ensure patient confidentiality and data anonymization.

## 5. Conclusions

Sequestrectomy is confirmed an effective treatment for MRONJ, achieving a high rate of healing and a relatively low recurrence rate. In this clinical audit, the addition of the PENTO protocol to antibiotic therapy did not result in a statistically significant improvement compared with antibiotic therapy alone, either in terms of complete healing or recurrence prevention. These findings should be interpreted with caution given the retrospective design, limited sample size, and other inherent limitations of a clinical audit. Further prospective, randomized studies with larger sample sizes and longer follow-up periods are needed to better define the role of the PENTO protocol, to evaluate safety profile and to identify specific patient subgroups that may benefit from this therapeutic approach.

## Figures and Tables

**Table 1:** Demographic and clinical characteristics of patient cohort (number of patients, n = 86).

Characteristics	Number (n) of Patients (%)
***Sex***	
Female	70 (81.4%)
Male	16 (18.6%)
***Age (years)***	
Mean (sd)	72.22 ± 13.17
Range	30–95
***Reason for using antiresorptive drug***	
Osteometabolic disease	30 (34.88%)
Oncological disease	55 (63.95%)
Not specified	1 (1.17%)
***Systemic concomitant drug therapy (additional risk factor for MRONJ)***	
Chemotherapy	8 (9.30%)
Corticosteroids	6 (6.97%)
Immunotherapy	14 (16.28%)
Hormone therapy	4 (4.65%)
Combination therapy	12 (13.95%)
No therapy	42 (48.85%)
***Trigger factor of the MRONJ***	
Extraction	25 (29.07%)
Implant	25 (29.07%)
None	36 (41.86%)
***Anatomical location***	
Mandible	56 (65.12%)
Maxilla	26 (30.23%)
Mandible and maxilla	4 (4.65%)
***MUCONN SCORE***	
1–5	47 (54.65%)
6–10	35 (40.70%)
11–15	3 (3.49%)
Not specified	1 (1.16%)
***MRONJ stage***	
0	1 (1.16%)
1	32 (37.21%)
2	41 (47.68%)
3	11 (12.79%)
Not specified	1 (1.16%)
***Antiresorptive drug associated with MRONJ***	
Alendronate	8 (9.32%)
Ibandronate	9 (10.46%)
Zoledronate	21 (24.42%)
Zoledronate + denosumab	2 (2.32%)
Denosumab	34 (39.53%)
Other bisphosphonates	12 (13.95%)
***Follow-up, in months***	
Mean (sd)	9.16 ± 11.19
Range	0–60

**Table 2:** Clinical characteristics of the two patient groups, i.e., PENTO (plus antibiotics) group (number of patients, n = 62) and antibiotics-only group (number of patients, n = 24).

Characteristics	PENTO (+Antibiotic) Group (n = 62)	Antibiotics-Only Group (n = 24)
***Sex***		
Female	48 (77.42%)	22 (91.67%)
Male	14 (22.58%)	2 (8.33%)
***Age (years)***		
Mean (±sd)	72.22 ± 13.17	64 ± 13.63
Range	30–95	54–74
***Reason for using antiresorptive drug***		
Osteometabolic disease	18 (29.03%)	12 (50.00%)
Oncological disease	44 (70.97%)	11 (45.83%)
Not specified	0 (0%)	1 (4.17%)
***Systemic concomitant drug therapy (additional risk factor for MRONJ)***		
Chemotherapy	5 (8.06%)	3 (12.50%)
Corticosteroids	2 (3.23%)	4 (16.67%)
Immunotherapy	12 (19.35%)	2 (8.33%)
Hormone therapy	3 (4.84%)	1 (4.17%)
Combination therapy	10 (16.13%)	2 (8.33%)
No therapy	30 (48.39%)	12 (50.00%)
***Trigger factor of the MRONJ***		
Extraction	25 (40.32%)	5 (20.83%)
Implant	25 (40.32%)	10 (41.67%)
None	36 (58.06%)	9 (37.50%)
***Anatomical location***		
Mandible	42 (67.74%)	14 (58.33%)
Maxilla	16 (25.81%)	10 (41.67%)
Mandible and maxilla	4 (6.45%)	0 (0%)
***MUCONN SCORE***		
1–5	34 (54.84%)	13 (54.17%)
6–10	25 (40.32%)	10 (41.67%)
11–15	3 (4.84%)	0 (0%)
Not specified	0 (0%)	1 (4.17%)
***MRONJ stage***		
0	0 (0%)	1 (4.17%)
1	26 (41.94%)	6 (25.00%)
2	27 (43. 55%)	14 (58.33%)
3	9 (14.52%)	2 (8.33%)
Not specified	0 (0%)	1 (4.17%)
***Antiresorptive drug associated with MRONJ***		
Alendronate	5 (9.32%)	3 (12.50%)
Ibandronate	6 (10.46%)	3 (12.50%)
Zoledronate	17 (24.42%)	4 (16.67%)
Zoledronate + denosumab	2 (2.32%)	0 (0%)
Denosumab	26 (39.53%)	8 (33.33%)
Other bisphosphonates	6 (13.95%)	6 (25.00%)
***Follow-up, in months***		
Mean (±sd)	9.16 ± 11.19	9.86 ± 11.80
Range	0–60	0.47–28

**Table 3:** Clinical outcomes among non-healed MRONJ sites (n = 13), considering the two groups, i.e., PENTO protocol combined with antibiotic therapy versus antibiotic therapy alone. Number of sites which improved, remained stable, or worsened are reported.

	PENTO Protocol + Antibiotic Therapy (Number of MRONJ Sites)	Antibiotic Therapy (Number of MRONJ Sites)
Improved	4	2
Worsened	2	0
Stable	4	1

**Table 4:** Clinical healing of MRONJ.

Healed MRONJ	Complete healing was defined as the absence of bone exposure, oral mucosal inflammation, infection, or clinical drainage for at least 3 months following sequestrectomy or spontaneous exfoliation of necrotic bone.
Stable MRONJ	A patient was considered “stable” when, at the last available follow-up visit, clinical evidence of MRONJ was still present, but the disease stage remained unchanged compared to the initial evaluation
Worsened MRONJ	A patient was considered “worsened” if, at the last available follow-up visit, clinical evidence of MRONJ was present and the disease had progressed to a more advanced stage compared to the initial diagnosis
Improved MRONJ	A patient was considered “improved” if, at the last available follow-up visit, clinical evidence of MRONJ was still present but the disease had regressed to a less advanced stage compared to the initial diagnosis

## Data Availability

The raw data supporting the conclusions of this article will be made available by the authors on request.

## Abbreviations

The following abbreviations are used in this manuscript:

MRONJ	Medication-related osteonecrosis of the jaws
AAOMS	American Association of Oral and Maxillofacial Surgeons
PENTO	Pentoxifylline and tocopherol
ORN	Osteoradionecrosis
